# Lacticaseibacillus parahuelsenbergensis sp. nov., Lacticaseibacillus styriensis sp. nov. and Lacticaseibacillus zeae subsp. silagei subsp. nov., isolated from different grass and corn silage

**DOI:** 10.1099/ijsem.0.006441

**Published:** 2024-07-02

**Keywords:** corn silage, grass silage, *Lacticaseibacillus parahuelsenbergensis*, *Lacticaseibacillus styriensis*, *Lacticaseibacillus zeae*, lactic acid bacteria, new species, new subspecies

## Abstract

Four rod-shaped, non-motile, non-spore-forming, facultative anaerobic, Gram-stain-positive lactic acid bacteria, designated as EB0058^T^, SCR0080, LD0937^T^ and SCR0063^T^, were isolated from different corn and grass silage samples. The isolated strains were characterized using a polyphasic approach and EB0058^T^ and SCR0080 were identified as *Lacticaseibacillus zeae* by 16S rRNA gene sequence analysis. Based on whole-genome sequence-based characterization, EB0058^T^ and SCR0080 were separated into a distinct clade from *Lacticaseibacillus zeae* DSM 20178^T^, together with CECT9104 and UD2202, whose genomic sequences are available from NCBI GenBank. The average nucleotide identity (ANI) values within the new subgroup are 99.9 % and the digital DNA–DNA hybridization (dDDH) values are 99.3–99.9 %, respectively. In contrast, comparison of the new subgroup with publicly available genomic sequences of *L. zeae* strains, including the type strain DSM 20178^T^, revealed dDDH values of 70.2–72.5 % and ANI values of 96.2–96.6 %. Based on their chemotaxonomic, phenotypic and phylogenetic characteristics, EB0058^T^ and SCR0080 represent a new subspecies of *L. zeae*. The name *Lacticaseibacillus zeae* subsp. *silagei* subsp. nov. is proposed with the type strain EB0058^T^ (=DSM 116376^T^=NCIMB 15474^T^). According to the results of 16S rRNA gene sequencing, LD0937^T^ and SCR0063^T^ are members of the *Lacticaseibacillus* group. The dDDH value between the isolates LD0937^T^ and SCR0063^T^ was 67.6 %, which is below the species threshold of 70 %, clearly showing that these two isolates belong to different species. For both strains, whole genome-sequencing revealed that the closest relatives within the *Lacticaseibacillus* group were *Lacticaseibacillus huelsenbergensis* DSM 11542*5* (dDDH 66.5 and 65.9 %) and *Lacticaseibacillus casei* DSM 20011^T^ (dDDH 64.1 and 64.9 %). Based on the genomic, chemotaxonomic and morphological data obtained in this study, two novel species, *Lacticaseibacillus parahuelsenbergensis* sp. nov. and *Lacticaseibacillus styriensis* sp. nov. are proposed and the type strains are LD0937^T^ (=DSM 116105^T^=NCIMB 15471^T^) and SCR0063^T^ (=DSM 116297^T^=NCIMB 15473^T^), respectively.

## Data Summary

Supplementary material and one figure are available at https://microbiology.figshare.comhttps://doi.org/10.6084/m9.figshare.26124445[1]

## Introduction

Silage is a crucial source of nutrition for ruminants, particularly cattle, as fresh feed is seasonal and therefore unavailable in winter. By ensiling fresh plant material such as maize, grass or cereals, the feed can be preserved. Aerobic and facultative anaerobic bacteria and yeasts utilize residual oxygen during silage fermentation. When oxygen levels drop, lactic acid bacteria take over and convert water-soluble carbohydrates into organic acids such as lactic acid, acetate and propionate (1,2-propandiol is a substrate for propionate formation), lowering the pH level. As a result, harmful bacteria and fungi are prevented from growing [2,5]. To further investigate and better describe the ensiling process, we started a screening for lactic acid bacteria in silage.

In 2020 the genus *Lactobacillus* was re-classified using phylogenomic approach [6]. According to the List of Prokaryotic names with Standing in Nomenclature (https://lpsn.DSMhttps://lpsn.DSMz.dehttps://lpsn.dsmz.de/), 29 species and two subspecies were validly published within the genus *Lacticaseibacillus* at the time of writing [7]. The *Lacticaseibacillus* species are characterized as Gram-positive, facultatively anaerobic and non-spore-forming microorganisms [6].

In 1959, Kuznetsov [8] proposed a new species, *Lactobacterium zeae* ATCC 15820^T^ (=DSM 20178^T^), isolated from corn-steep liquor. Due to high DNA similarity (82 %) to the neotype strain of *Lactobacillus casei* ATCC 393^T^ (=DSM 20011^T^) as well as good agreement in DNA base composition, *Lactobacterium zeae* ATCC 15820^T^ was assigned to the species *Lactobacillus casei* in 1973 by Mills and Lessel [9]. As the genotypic approach has made phenotypic and genotypic distinctions more precise, *Lacticaseibacillus casei* was reclassified in 2020 into two species again: *Lacticaseibacillus zeae* and *Lacticaseibacillus casei* [10].

Sequencing the 16S rRNA gene is a first step towards identifying the species or even subspecies of bacteria. Due to the high sequence similarity of this gene in many *Lacticaseibacillus* species (>99.7 % for *Lacticaseibacillus casei* and *Lacticaseibacillus zeae*), this method is not always applicable [10,11]. Genome sequencing followed by reconstruction of a phylogenetic tree using >90 housekeeping genes is a standard method to classify species or subspecies [12,15].

Using whole genome sequencing and subsequent genotypic characterization, many new *Lacticaseibacillus* species have recently been isolated and characterized. *Lacticaseibacillus absinus* [16], *Lacticaseibacillus kribbianus* [17], *Lacticaseibacillus chiayiensis* [18], *Lacticaseibacillus saniviri* [19] and *Lacticaseibacillus parakribbianus* [20] were isolated from cecum or faeces of animals. *Lacticaseibacillus huelsenbergensis* [15] was isolated from grass and corn silage, showing a wide distribution of *Lacticaseibacilli*.

Here we present the isolation and polyphasic characterization of two novel *Lacticaseibacillus* species (*Lacticaseibacillus parahuelsenbergensis* and *Lacticaseibacillus styriensis*) and a novel subspecies of *L. zeae, Lacticaseibacillus zeae* subsp. *silagei*.

## Methods and results

### Isolation

Lactic acid bacteria were isolated from different silage samples. Fresh corn was harvested from a local field in Großotten, Austria (latitude 48.63809° N, longitude 14.979151° E), from Kapfenberg, Austria (latitude 47.45808° N, longitude 15.34139° E) and from Haag, Austria (latitude 48.11670° N, longitude 14.36281° E), while grass material was gathered from a field in Güssing, Austria (latitude 47.08785° N, longitude 16.30298° E). Furthermore, the fresh material was chopped into 30–50 mm long pieces and stored without silage additives in a small-scale fermentation system as described previously [5]. Strains were isolated from grass or corn silage by making an extract with 0.9 % NaCl and plating serial dilutions on deMan, Rogosa and Sharpe (MRS; Lactan) agar plates. After incubation for 48 h at 37 or 45 °C under semi-anaerobic conditions in an anaerobic box (Lava and anaeroGen bags from Thermo Fisher Diagnostics BV), bacterial colonies with different appearance were isolated, cultivated in MRS broth for 24 h, portioned and stored as glycerol stocks at −80 °C for further investigation. Three isolates were obtained from different corn silage, one as EB0058^T^ (Großotten, Austria), another as SCR0080 (Kapfenberg, Austria) and a third one as SCR0063^T^ (Haag, Austria). One bacterial strain was isolated from grass silage (Güssing, Austria) and named LD0937^T^.

### Identification

The 16S rRNA gene was sequenced in order to identify isolated colonies. The DNeasy Kit (Qiagen) was used to extract the bacterial genomic DNA according to the manufacturer’s instructions. The DreamTaq Green PCR Master Mix (Fermentas, Art. Nr. K1082) and universal primers 27F and 1492R were used to amplify the 16S rRNA gene, as previously reported [20,21]. blast analysis of the 16S rRNA gene sequence was performed using the GenBank database (https://blast.ncbi.nlm.nih.gov/BlastAlign.cgi). Results of the blast analysis showed a high similarity of EB0058^T^ and SCR0080 to the *Lacticaseibacillus zeae* group (99.9 %–100 %), whereas LD0937^T^ and SCR0063^T^ could not be clearly assigned to a *Lacticaseibacillus* species (Table S1, available in the online version of this article). Sequence similarity of the 16S rRNA genes within the *Lacticaseibacillus* group is reported to be too high to guarantee a species identification using this method [10,11, 13, 22]. Due to the high sequence similarity of EB0058^T^, LD0937^T^ and SCR0063^T^ to the type strains *Lacticaseibacillus casei* DSM 20011^T^ (99.6 %/99.5 %/99.3 %), *Lacticaseibacillus chiayiens*is NBRC 112906^T^ (99.6 %/99.3 %/99.3 %), *Lacticaseibacillus huelsenbergensis* DSM 115425^T^ (99.6%/99.5%/99.4 %), *Lacticaseibacillus zeae* DSM 20178^T^ (99.9 %/99.3 %/99.3 %), *Lacticaseibacillus paracasei* subsp. *tolerans* DSM 20258^T^ (99.4 %/99.3 %/99.2 %) *Lacticaseibacillus paracasei* subsp. *paracasei* DSM 5622^T^ (99.2%/99.0%/98.8%) and *Lacticaseibacillus rhamnosus* DSM 20021^T^ (99.0%/98.7 %/98.6 %), they could not be clearly assigned to a species, necessitating further analysis.

The reference strains used in this work (*L. zeae* DSM 20178^T^, *L. huelsenbergensis* DSM 115425^T^, *L. casei* DSM 20011^T^*, L. paracasei* subsp. *paracasei* DSM 5622^T^, *L. paracasei* subsp. *tolerans* DSM 20258^T^ and *L. rhamnosus* DSM 20021^T^) were obtained from the DSMZ – German Collection of Microorganisms, Braunschweig, Germany. *L. chiayiens*is NBRC 112906^T^ was acquired from the Biological Resource Center, NITE (NBRC) in Tokyo, Japan.

DNA fingerprinting by amplification of repetitive genome sequences was often used to determine the species or subspecies of bacteria until genome sequencing became available [15,16, 23, 24]. The method remains a rapid and effective means of distinguishing between species. The PCR reactions were performed with the novel isolates and reference strains mentioned above, using the primers (GTG)_5_, ERIC1R and ERIC2 and BOXA1R as described by Versalovic *et al*. [25]. The fingerprinting PCR could clearly differentiate the novel isolates from the reference strains (Fig. S1). *L. zeae* DSM 20178^T^, which had the highest 16S rRNA similarity, showed clear differences in the banding pattern, highlighting the need for further analysis. Therefore, the whole genomes of EB0058^T^, SCR0080, LD0937^T^ and SCR0063^T^ were sequenced and assembled.

### Genome sequencing

The whole genome was sequenced at IIT Biotech GmbH (Bielefeld, Germany) with a hybrid strategy using Oxford Nanopore Technologies and Illumina Technologie. Genomic DNA of strains of EB0058^T^, SCR0080, LD0937^T^ and SCR0063^T^ was isolated via the NucleoSpin Microbial DNA kit (Macherey-Nagel). Long and short DNA reads were generated by Nanopore and Illumina sequencing, respectively. For library preparation, the TruSeq DNA PCR-free high-throughput library prep kit (Illumina) and the SQK-LSK112 (for EB0058^T^, LD0937^T^) or SQK-LSK114 (for SCR0080, SCR0063^T^) sequencing kits (Oxford Nanopore Technologies) were used without prior shearing of the DNA. To generate the short reads, a 2×300-nucleotide run (MiSeq reagent kit v3, 600 cycles) was executed. The long reads were generated on a MinION platform using R10.4.1 (for SCR0080) or R9.4.1 (for EB0058^T^) flow cells and on a GridION platform using R10.4 (for LD0937^T^) and R10.4.1 (for SCR0063^T^) flow cells. Base calling and demultiplexing were performed using Guppy version 6.2.11 (for LD0937^T^) and Guppy version 6.3.9 (for EB0058^T^, SCR0080 and SCR0063^T^) with the super-accurate base-calling model. Assemblies were done using Flye version 2.9 [26] for the Nanopore long read data and Newbler version 2.8 [27] for the Illumina short-read data. After polishing of the Flye-based assembly using Medaka version 1.6.1 [28], Pilon version 1.22 [29] and Bowtie2 [30] for mapping, the respective Flye and Newbler assemblies were combined in Consed version 28.0 [31]. The resulting single contigs representing the circular genomes were annotated using the PGAP pipeline versions 2022-12-13 (LD0937^T^) and 2023-05-17 (EB0058^T^, SCR0080 and SCR0063^T^) [32,33], and prokka version 1.14.6 [34] and are available at GenBank, accessions GCA_030770335.1 (EB0058^T^), GCA_030770315.1 (SCR0080) GCA_030770265.1 (LD0937^T^) and GCA_030770295.1 (SCR0063^T^).

The genome sizes of EB0058^T^, SCR0080, LD0937^T^ and SCR0063^T^ were 3.05, 3.07, 3.06 and 3.13 Mb, coverage values were 941×, 281×, 525× and 381×, respectively, and the molar G+C content was 48.0 mol%. The coding sequence values were 2777, 2824, 2792 and 2096 the number of total RNA was 108, 107, 103 and 104, respectively (Table S2).

The completeness of the genome assembly was analysed using CheckM [35]. In addition, the 16S rRNA sequences were aligned with the genome sequences to verify the assemblies.

### Genotypic characterization

The whole genome sequences of EB0058^T^, SCR0080, LD0937^T^ and SCR0063^T^ were submitted to TYGS [36,37]. Strains EB0058^T^ and SCR0080 were identified as *Lacticaseibacillus zeae*, whereas LD0937^T^ and SCR0063^T^ were identified as representing two potential novel species. Reference strains for bioinformatic analysis are all valid *Lacticaseibacillus* type strains currently available at LPSN, plus *Lacticaseibacillus zeae* genomes submitted to GenBank (*L. zeae* CECT9104 (GCF_900492555.1), *L. zeae* FBL8 (GCF_018363055.1), *L. zeae* KCTC 3804^T^ (GCF_000260435.1), *L. zeae* UHGG_MGYG-HGUT-02383 (GCF_902386575.1), * L. zeae* CRBIP24.58 (GCF_005796005.1), *L. zeae* UD2202 (GCF_028878215.1). The genome sequence and annotation files were downloaded from the RefSeq database (https://www.ncbi.nlm.nih.gov/refseq/). TYGS reconstructed a proteome-based phylogenetic tree showing that LD0937^T^ and SCR0063^T^ form distinct clades that are closely related to *Lacticaseibacillus casei* and *Lacticaseibacillus huelsenbergensis* (Fig. S2). While isolates EB0058^T^ and SCR0080 were identified as *Lacticaseibacillus zeae*, the phylogenomic analysis shows clustering into two distinct clades: one group containing EB0058^T^, SCR0080, UD2202 and CECT9104, which is distinguished from the other *L. zeae* strains used in this study.

Chun *et al*. proposed the computation of the overall genome related index in order to detect novel species [38]. They suggest the species boundary to be an average nucleotide identity (ANI) value between 95–96 % or a digital DNA–DNA hybridization (dDDH) value of 70 %. According to Meier-Kolthoff *et al*., dDDH analyses outperform ANI analyses [36]. The Genome-to-Genome Distance Calculator was used to calculate dDDH values with the recommended formula 2 [37,39]. The ANI values were calculated using the JSpecies Web Server with blast+ mode [40]. The two novel *L. zeae* isolates EB0058^T^ and SCR0080 have an ANI value of 99.9 % and a dDDH value of 99.6 %, indicating that both strains belong to the same species (Tables 1 and S3). Isolates LD0937^T^ and SCR0063^T^ have a dDDH value of 67.6 % and an ANI value of 95.9 % (Table 1). This suggests that both organisms belong to distinct species. Isolates EB0058^T^, SCR0080, LD0937^T^ and SCR0063^T^ could be clearly distinguished from *L. huelsenbergensis* DSM 115425^T^, *L. casei* DSM 20011^T^*, L. chiayiens*is NBRC 112906^T^, *L. paracasei* subsp. *paracasei* DSM 5622^T^, *L. paracasei* subsp. *tolerans* DSM 20258^T^ and *L. rhamnosus* DSM 20021^T^, whereas two groups of *L. zeae* strains were identified. The *L. zeae* strains EB0058^T^, SCR0080, UD2202 and CECT9104 belong to one group with ANI and dDDH values>99.9 % and >99.3 %, respectively, within the group. However, the values compared to the second group (including the *L. zeae* strains DSM 20178^T^, KCTC3804^T^, CRBIP24.58, FBL8 and UHGG_MGYG-HGUT-02383) were 96.3 and 71 %, respectively. Meier-Kolthoff *et al*. [41] proposed a dDDH value of 79–80 % as the threshold for delineating subspecies, which indicates two subspecies within the *Lacticaseibacillus zeae* species. This novel subspecies is further distinguished from its closest relatives using additional genotypic methods such as the core genome phylogenetic tree or Rep-PCR, and phenotypic characterization of carbohydrate metabolism and physiological characteristics.

**Table 1.** Average nucleotide identity (ANI) values (top-right values) and digital DNA–DNA hybridization (dDDH; formula 2) values (bottom-left values) for strains EB0058^T^, SCR0080, LD0937^T^, SCR0063^T^ and related species

**Fig. 1.**
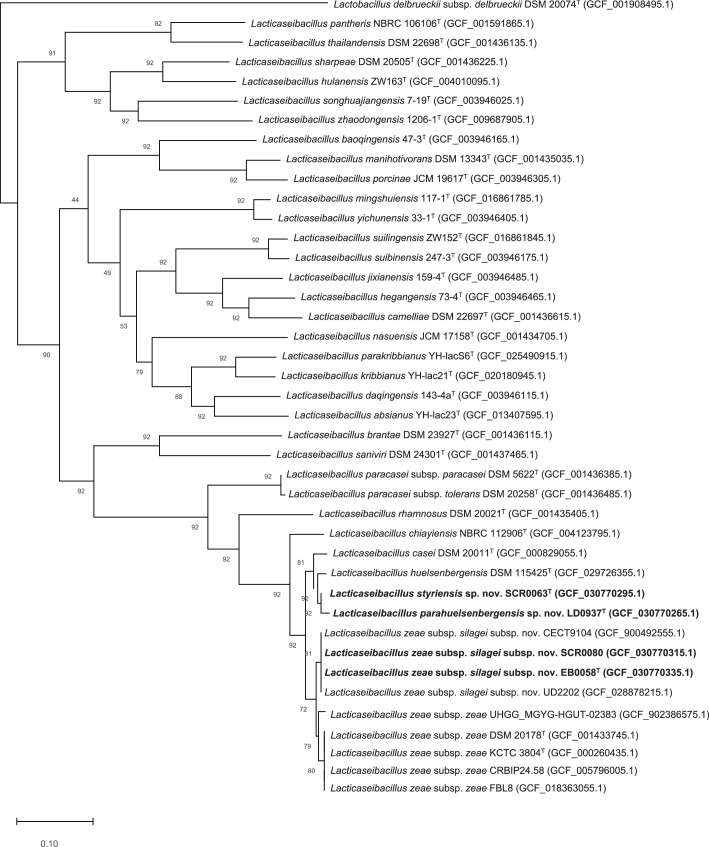
Phylogenetic analysis of the novel isolates EB0058^T^, SCR0080, LD0937^T^ and SCR0063^T^ core genomes using the UBCG tool. Reference strains are all valid *Lacticaseibacillus* type strains currently available from LPSN, plus *L. zeae* genomes downloaded from NCBI GenBank. *Lactobacillus delbrueckii* subsp. *delbrueckii* DSM 20074^T^ served as the outgroup. Gene support index indicates how many genes support the branch in the concatenated phylogenetic tree. Bar, 0.1 substitutions per nucleotide position.

For further analysis of the novel strains within the genus *Lacticaseibacillus*, an additional core gene phylogenetic tree was reconstructed with all previously mentioned reference strains (Table S1) using the UBCG tool version 3.0 [42] and default options (Fig. 1). The core gene phylogenetic tree was visualized using mega 11 software (version 11.0.3) [43]. According to the analysis of the core genome, the genomes of EB0058^T^, SCR0080, UD2202 and CECT9104 form a distinct clade within the *L. zeae* group, while isolates LD0937^T^ and SCR0063^T^ form two distinct clusters within the *L. casei* group (Fig. 1). Thus, the isolates can be identified as representing novel species (*L. parahuelsenbergensis* LD0937^T^ and *L. styriensis* SCR0063^T^) and a novel subspecies of *L. zeae (L. zeae* subsp. *silagei* EB0058^T^ and *L. zeae* subsp. *silagei* SCR0080).

To distinguish these novel species using PCR methods, housekeeping genes with variations were amplified. The housekeeping genes *dnaK*, *rpoA*, *yycH*, *pheS*, *mutL*, *rpoA* and *argS* are among the alternative phylogenetic markers that are frequently employed to identify species [13,14, 18, 44,46]. The sequence similarity of the housekeeping genes *rpoA* (939 bp) and *argS* (1683 bp) of EB0058^T^ and SCR0080, *rpoB* (3579 bp) of SCR0063^T^ and the *pheS* gene (1047 bp) of LD0937^T^ were calculated using megablast (Table S1). The sequence similarity between strains EB0058^T^, SCR0080, UD2202 and CECT9104 is 100 % for both genes (*rpoA* and *argS*), while the similarity for the genes of the other *L. zeae* isolates is <99.4 % for the *rpoA* gene and <98.7 % for the *argS* gene (Table S1). The sequence similarities of the genes compared to the reference strains were <97% for the *pheS* gene of LD0937^T^ and <98.5 % for the *rpoB* gene of SCR0063^T^. As previously published [15,18, 46], primers were designed to specifically amplify the housekeeping gene of the newly discovered species and subspecies after identifying gene regions of variability: Lzsil_*argS*_fw1 (5′-CGGCAATGCCAAAATGGGGA-3′) and Lzsil_*argS*_rev2 (5′-CAAGCGTCTTAACCACGCTGT-3′) for amplification of the *argS* gene of the novel subspecies, *pheS*_fw3 (5′-AGACCATCCGGCACGC-3′) and *pheS*_rev4 (5′-ATGCTGACACATGGCCAGTAAG-3′) specifically amplifying the *pheS* gene of LD0937^T^, and *rpoB*_fw2 (5′-GAACTCCCCACTGAACGATGAT-3′) and *rpoB*_rev4 (5′-CATCAACGTCAACATGATCGC-3′) for the amplification of the *rpoB* gene of SCR0063^T^. The PCR was performed using 25 µl in total, containing 1 U Dream Taq polymerase, 2 mM MgCl_2_, 0.2 mM dNTP and 0.4 µM of each primer. The PCR cycling programme was 95 °C for 5 min, 25 cycles of 95 °C for 30 s., 55 or 60 °C for 30 s and 72 °C for 1 min, and a final elongation cycle at 72 °C for 5 min. The amplified PCR product consisted of 499 bp (*argS*), 270 bp (*pheS*) and 463 bp (*rpoB*). To test the primer’s specificity, 36 other *Lactobacillus* species, including those that are closely related, were utilized. However, when amplifying the *argS* gene, only the two isolates EB0058^T^ and SCR0080 showed evidence of the DNA fragment, while all other reference strains, including the *L. zeae* type strain DSM 20178^T^ did not show any fragments (Fig. S3). Only LD0937^T^ showed evidence of the *pheS* DNA fragment, while the *rpoB* gene fragment was only detected in SCR0063^T^; all other reference strains did not show any fragments (Figs S4 and S5).

### Functional genomics

The core and accessory genes of the novel strains and their closest relatives were calculated using the Roary pan genome pipeline version 3.13.0 [47] after annotation with the prokka pipeline version 1.14.6 [34]. The predicted coding sequences (CDS) of the novel strains and their closed relatives were analysed using the kegg Automatic Annotation Server (kaas) version 2.1 [48] in order to confirm the results.

The number of CDS of various kegg pathways was similar for EB0058^T^ and SCR0080, while both strains exhibit clear distinctions from * L. zeae* DSM20178^T^ in the kegg orthologue ABC transporter, carbon metabolism, metabolic pathways especially the pentose phosphate pathway and galactose metabolism (see Table S4). The Roary pan genome analysis revealed that isolates EB0058^T^ and SCR0080, as well as the other two genome sequences from the proposed new subspecies in group A (Table 1), contain the *araA* gene for the l-arabinose isomerase. On the other hand, *L. zeae* DSM 20178^T^, as well as all the other strains in group B (proposed *L. zeae* subsp. *zeae*), are lacking the gene *araA*. These results were confirmed with the kegg tool and phenotypically in metabolic experiments. This distinction permits the two proposed subspecies to be effectively delineated, *L. zeae* subsp. *silagei* with the type strain EB0058^T^ and *L. zeae* subsp. *zeae* with the type strain DSM 20178^T^.

Furthermore, strains LD0937^T^ and SCR0063^T^ could be distinguished from all their closely related type strains, because only these two strains possess the *araA* gene and therefore an l-arabinose isomerase. Roary analysis showed, that only SCR0063^T^ has the sequence for an α-mannosidase. This enzyme is responsible for metabolising methyl α-d-mannopyranoside, a model substrate, also used in the API system. This result was also verified with the kegg tool, where only SCR0063^T^ was found to possess an α-mannosidase (EC.3.2.1.24), in contrast to LD0937^T^ and all related *Lacticaseibacillus* type strains. The differential utilization of the substrates was validated through phenotypic analysis in metabolic experiments. The number of CDS of various kegg pathways (for example: ABC transporter, biosynthesis of secondary metabolites, metabolic pathways, pentose phosphate pathway and phosphotransferase system) differs between LD0937^T^ and SCR0063^T^. Furthermore, both strains exhibit clear distinctions from *L. huelsenbergensis* DSM115425^T^ in the kegg orthologue biosynthesis of secondary metabolites, fructose and mannose metabolism, metabolic pathways, phosphotransferase system and starch and sucrose metabolism (see Table S4). Among other things, this fact helps to differentiate between the two novel proposed species with the type strains LD0937^T^and SCR0063^T^.

The whole genome sequences were analysed for the presence of antimicrobial resistance (AMR) genes. For this purpose, whole genomic DNA of the strains were compared to entries in the ResFinder database using the bioinformatic tool ResFinder (version 4.5.0.) [49,51]. For the blast-based search, ResFinder’s default settings for minimum DNA identity were changed to 80 % and coverage was set to 5 %, since the European Food Safety Authority statement on the requirements for whole genome sequence analysis of microorganisms intentionally used in the food chain [52] recommends searching the AMR genes with a minimal coverage. No AMR genes were identified in the genome sequences of EB0058^T^, SCR0080, LD0937^T^ and SCR0063^T^.

### Phenotypic and chemotaxonomic characterization

Prior to morphological, phenotypic or chemotaxonomic characterization, the novel strains and all reference bacteria were cultivated on MRS agar plates at 37 °C for 24 h under semi-anaerobic conditions (anaerobic box from Lava and anaeroGen bags from Thermo Fisher Diagnostics). Each experiment was conducted three times. Gram staining was evaluated in accordance with the manufacturer’s instructions using Gram-staining solutions from Merck. A phase-contrast microscope (Leica DM 2500 LED) was used to determine spore development and Gram staining. Under aerobic and anaerobic circumstances, gas formation from glucose was examined in MRS broth at 37 °C using inverted Durham tubes.

The Enzytec d/l lactic-acid kit from r-Biopharm was used to determine the enzymatic production of lactic acid isomers. Growth tests were conducted in MRS broth with a range of parameters, including temperature (5, 10, 20, 30, 37, 45 and 55 °C), pH (pH 3.0, 3.5, 4.0, 4.5, 5.0, 5.5, 7.0, 7.5, 8.0, 8.5, 9.0, 9.5, 10.0 and 10.5), NaCl salt concentrations (4–10 % w/v) and KCl salt concentrations (6.0, 8.4 and 10.8 % w/v). Using the commercially available API 50 CH and API ZYM systems (bioMèrieux), the capacity for carbohydrate metabolism and the enzymatic activity was measured. The respiratory quinones were established since the oxidase test was negative. Additional phenotypic and biochemical analysis (such as measuring catalase activity, metabolic experiments and cell motility) were carried out according to established protocols [53]. A haemolysis test on blood agar plates was additionally performed as previously described [54,56].

Whole cell fatty acids are analysed after conversion into fatty acid methyl esters by saponification, methylation and extraction following the protocol of Sasser [57]. The fatty acid methyl esters mixtures are separated by gas chromatography and detected by a flame ionization detector. In subsequent analysis, fatty acids are identified by a GC-MS run, on an Agilent GC-MS 7000D system [58]. Peaks were identified based on retention time and mass spectra.

Polar lipids were extracted from freeze-dried cell material using a chloroform–methanol–0.3 % aqueous NaCl solution, polar lipids were recovered into the chloroform phase [59] and separated by two-dimensional silica gel TLC. The first direction was developed in chloroform–methanol–water and the second in chloroform–methanol–acetic acid–water. Total lipid material was detected using molybdatophosphoric acid and specific functional groups detected using spray reagents specific for defined functional groups [60].

Analysis of cellular fatty acids, peptidoglycan structure, respiratory quinones and polar lipids was carried out by DSMZ Services, Leibniz-Institut DSMZ – Deutsche Sammlung von Mikroorganismen und Zellkulturen GmbH, Braunschweig, Germany.

Growth and distinctive features among strains EB0058^T^, SCR0080 and the closest phylogenetically related type strain *L. zeae* DSM 20178^T^ can be found in Table 2. Isolates EB0058^T^ and SCR0080 show totally different growth behaviours from the type strain *L. zeae* DSM 20178^T^. Both isolates can grow at 5 °C and with 9 % w/v NaCl and 8.4 % w/v KCl, but they cannot grow at pH 10, in contrast to their nearest phylogenetic relative. EB0058^T^ and SCR0080 could produce acid from l-arabinose, sorbitol, maltose and d-arabitol and they are positive for α-glucosidase and negative for alkaline phosphatase and cysteine arylamidase, which differed from the characteristics of the nearest phylogenetic neighbour *L. zeae* DSM 20178^T^. These results were also confirmed in metabolic experiments and with functional genomics. *L. zeae* DSM 20178^T^ is not able to metabolize l-arabinose, because it lacks the *araA* gene and therefore does not possess an l-arabinose isomerase, in contrast to both novel isolates EB0058^T^ and SCR0080. Furthermore, all the strains of group A possess the l- arabinose isomerase (EC 5.3.1.4), whereas this enzyme is absent in all strains of group B (Table 1). This is the reason why only group A strains are capable of metabolizing l-arabinose to lactic acid. This distinction permits the two proposed subspecies to be effectively delineated, *L. zeae* subsp. *silagei* with the type strain EB0058^T^ and *L. zeae* subsp. *zeae* with the type strain DSM 20178^T^.

**Table 2.** Differential phenotypic and growth characteristics of the novel strains and their phylogenetically closest related type strain *L. zeae* DSM 20178^T^ Strains: 1, EB0058^T^; 2, SCR0080; 3, *L. zeae* DSM 20178^T^; +, − and w represent good, no, weak growth or reaction, respectively.

Characteristic	1	2	3
**Growth at:**			
5 °C	+	+	−
50 °C	+	+	+
pH 3.5	+	+	+
pH 10	−	−	+
9 % NaCl	+	+	−
8.4 % KCl	+	+	w
**Enzymatic activity:**			
Oxidation of glucose	+	+	−
Alkaline phosphatase	−	−	w
Cystine arylamidase	−	−	+
α- Glucosidase	+	+	−
**Acid production from:**			
l-Arabinose	+	+	−
Sorbitol	+	+	−
Maltose	+	+	−
d-Arabitol	+	+	−

The results of the cellular fatty acid composition are shown in Table 3. The ratios of the fatty acids between the two strains EB0058^T^ and SCR0080 were very similar. The dominant whole- cell fatty acids (mean value >10 % of whole fatty acids) of EB0058^T^ and SCR0080 were C_16 : 0_ (14.6 and 17.6 %), summed feature 7 (16.1 and 18.4 %) and summed feature 8 (27.2 and 26.2 %). The cellular fatty acid composition further indicates that both strains can be clearly distinguished from the closely related type strain *L. zeae* DSM 20178^T^, which shows different percentages in almost all fatty acid contents. In detail, dominant fatty acids of *L. zeae* DSM 20178^T^ were C_18 : 1_ ω9*c* (42.2 %), summed feature 7 (24.0 %) and C_16 : 0_ (10.0 %). Furthermore, totally different values were obtained for summed feature 3 (1.6 %) and summed feature 8 (6.3 %).

**Table 3.** Cellular fatty acids of the novel strains and the closest related species Strains: 1, EB0058^T^; 2, SCR0080; 3, *L. zeae* DSM 20178^T^; 4, LD0937^T^; 5, SCR0063^T^; 6*, L. huelsenbergensis* DSM 115425^T^; 7, *L. casei* DSM 20011^T^; 8, *L. chiayiensis* NBRC112906^T^; 9, *L. paracasei* subsp. *paracasei* DSM 5622^T^; 10, *L. rhamnosus* DSM 20021^T^. Values are percentage of total fatty acid detected. nd, not detected. Fatty acids present >10 % are indicated in bold. Fatty acids present <1 % are not shown in this table.

Fatty acid	1	2	3	4	5	6	7	8*	9*	10*
C_14 : 0_	2.7	2.9	4.7	**10.7**	8.4	6.1	6.0	2.0	8.2	3.8
C_16 : 0_	**14.6**	**17.6**	10.0	**13.2**	**13.5**	**14.0**	**14.2**	**36.1**	**41.1**	**37.2**
C_18 : 0_	1.6	1.9	1.2	0.8	1.0	1.3	0.5	3.5	2.2	3.7
C_18 : 1_ ω9*c*	9.4	7.9	**42.2**	**40.3**	**25.7**	**23.3**	**12.0**	**26.9**	**20.2**	4.0
Summed feature 3†	7.1	6.6	1.6	3.2	3.0	2.0	**10.3**	1.5	7.8	5.5
Summed feature 7†	**16.1**	**18.4**	**24.0**	**23.4**	**40.1**	**38.8**	**27.8**	**17.9**	**nd**	**23.3**
Summed feature 8†	**27.2**	**26.2**	6.3	7.3	6.8	6.8	**17.9**	**10.0**	**12.4**	**17.9**

a *Data from Huang, Chen *et al*. [10].

b †Summed feature 3:, C_16 : 1_ ω7*c*/C_16 : 1_ ω6*c*;. Summed feature 7:, C_19 : 1 _ω6*c* and/or C_19 : 1_cyclo ω10*c*, C_19 : 0_cyclo ω8*c* and / or unknown fatty acid (ECL 18.846);. Summed feature 8:, C_18 : 1_ ω7*c* or C_18 : 1_ ω6*c.*

The polar lipids of EB0058^T^ included diphosphatidylglycerol, phosphatidylglycerol, one unidentified aminophospholipid, one unidentified aminolipid, seven unidentified glycolipids and three unidentified lipids (Fig. S6A). The polar lipids of SCR0080 were very similar to EB0058^T^, they contained two unidentified amino phospholipids, but no unidentified amino lipid and one more unidentified glycolipid instead of an unidentified lipid (Fig. S6B). In contrast to *L. zeae* DSM 20178^T^, which contained neither an unidentified amino lipid nor unidentified lipids, as well as nine unidentified glycolipids (Fig. S6C). The polar lipid results showed that the two novel strains contained different polar lipids, but also had similar polar lipid compounds (diphosphatidylglycerol and phosphatidylglycerol) compared to the type strain *L. zeae* DSM 20178^T^.

Based on this polyphasic approach, using genomic, phylogenetic, phenotypic and chemotaxonomic analyses, EB0058^T^ and SCR0080 could be clearly distinguished from their closest relatives. Thus, strains EB0058^T^ and SCR0080 should be classified as belonging to a novel subspecies within the species *L. zeae* named *Lacticaseibacillus zeae* subsp. *silagei* for which the strain EB0058^T^ (=DSM 116376^T^=NCIMB 15474^T^) should be the type strain. Additionally, some of the publicly available genomes labelled as * L. zeae*, such as CECT9104 and UD2202, should be transferred to the new subspecies. According to Rule 40b of the Bacteriological Code [61], the description of a new subspecies creates automatically the subspecies *L. zeae* subsp. *zeae* with the type strain DSM 20178^T^ (=ATCC 15820^T^=RIA 482^T^=CCUG 35515^T^=KCTC 3804^T^=JCM 11302^T^=BCRC 17942^T^=LMG 17315^T^=NCIMB 9537^T^).

Table 4 displays the growth and distinguishing characteristics of the strains LD0937^T^, SCR0063^T^ and their closest related type strains. Strain LD0937^T^ showed weak growth at pH 3.5 and with 8.4 % KCl and good growth at pH 10 and with 8 % w/v NaCl in contrast to the closest related strains *L. huelsenbergensis* DSM 115425^T^ and *L. casei* DSM 20011^T^. Acid is produced from l-arabinose, l-sorbose and sucrose, whereas no acid is produced from dulcitol and turanose, unlike the nearest phylogenetic neighbours. SCR0063^T^ showed weak growth at 50 °C and good growth at pH 10 and with 8.4 % w/v KCl, compared to *L. huelsenbergensis* DSM 115425^T^. Furthermore, SCR0063^T^ is able to metabolize l-arabinose, methyl α-d-mannoside and maltose, but not dulcitol and melezitose in contrast to *L. huelsenbergensis* DSM 115425^T^ and *L. casei* DSM 20011^T^. LD0937^T^ and SCR0063^T^ are able to metabolize l-arabinose into lactic acid, because they possess the *araA* gene and therefore an l-arabinose isomerase, in contrast to all their closely related type strains. To distinguish between the two strains LD0937^T^ and SCR0063^T^ and all closely related type strains, it should be noted that only SCR0063^T^ has an α-mannosidase and can metabolize methyl α-d-mannopyranoside, a model substrate, also used in the API system. These results were also confirmed in metabolic experiments and with functional genomics. Therefore, metabolic experiments of LD0937^T^, SCR0063^T^ and all closely related *Lacticaseibacillus* type strains with this substrate were performed. As expected, only SCR0063^T^ was able to metabolize methyl α-d-mannopyranoside.

**Table 4.** Differential phenotypic and growth characteristics between the strains LD0937^T^ and SCR0063^T^ and phylogenetically related type strains Strains: 1, LD0937^T^; 2, SCR0063^T^; 3*, L. huelsenbergensis* DSM 115425^T^; 4, *L. casei* DSM 20011^T^; 5, *L. zeae* DSM 20178^T^; 6, *L. chiayiensis* NBRC112906^T^; 7, *L. paracasei* subsp. *paracasei* DSM 5622^T^; 8, *L. rhamnosus* DSM 20021^T^. nd: not determined; +, −, w: good, no, weak growth or reaction, respectively.

Characteristic	1	2	3	4	5	6	7	8
**Growth at/with:**								
10 °C	+	+	+	w	w	w	w	+
50 °C	+	w	+	+	+	+	w	+
pH 3.5	w	+	+	−	+	+	w	w
pH 10	+	+	−	−	+	nd	nd	nd
8 % NaCl	+	w	w	−	+	w	w	+
8.4 % KCl	w	+	w	−	w	+	w	w
**Enzymatic activity:**								
Alkaline Phosphatase	−	w	−	w	w	+	−	−
α-Galactosidase	w	+	w	+	w	+	−	−
α-Glucosidase	−	+	+	−	−	+	+	+
*N*-Acetyl-β-glucosaminidase	w	−	−	w	w	+	w	w
**Acid production from:**								
d-Arabinose	+	+	+	−	+	−	−	−
l-Arabinose	+	+	−	−	−	−	−	−
Adonitol	+	+	+	−	−	+	−	−
l-Sorbose	+	−	−	−	−	−	−	+
Ribose	+	+	+	−	+	+	+	+
Rhamnose	+	+	+	−	+	+	−	+
Dulcitol	−	−	+	−	−	+	−	−
Sorbitol	+	+	+	−	−	+	−	+
Methyl α-d-mannoside	−	+	−	−	−	−	−	−
Maltose	−	w	−	−	+	−	+	+
Lactose	+	+	+	+	−	−	+	+
Sucrose	+	+	−	−	+	−	+	−
Melezitose	+	−	+	+	+	−	+	+
Turanose	−	+	+	−	−	−	+	+
l-Fucose	+	+	+	−	+	+	−	−
l-Arabitol	+	+	+	−	−	−	−	−

The enzyme activity from LD0937^T^ was positive for *N*-acetyl-β-glucosaminidase and negative for α-glucosidase, while SCR0063^T^ showed good α-galactosidase activity and alkaline phosphatase activity, which differed partially from the characteristics of the nearest phylogenetic relatives and from each other.

Table 3 displays the results of the cellular fatty acid composition. The dominant whole-cell fatty acids (mean value >10 % of whole fatty acids) of LD0937^T^ were C_14 : 0_, C_16 : 0_, C_18 : 1_ ω9*c* and summed feature 7. For SCR0063^T^ they were C_16 : 0_, C_18 : 1_ ω9*c* and summed feature 7. The fatty acid ratios of trains LD0937^T^ and SCR0063^T^ showed differences in terms of C_18 : 1_ ω9*c* (40.3 and 25.7 %) and summed feature 7 (23.4 % and 40.1 %). Both strains showed higher C_14 : 0_ (10.7 and 8.4 %) and C_18 : 1_ ω9*c* (40.3 and 25.7 %) values than *L. huelsenbergensis* DSM 115425^T^ (6.1 and 23.3 %). The cellular fatty acid compositions of the three above mentioned strains are similar but there are clear differences in the proportions and values of C_14 : 0_, C_18 : 1_ ω9*c* and summed feature 7. Furthermore, LD0937^T^ and SCR0063^T^ have significantly different fatty acid ratios compared to *L. casei* DSM 20011^T^. They had different C_18 : 1_ ω9*c*, summed feature 3, summed feature 7 and summed feature 8 ratios compared to *L. casei* DSM 20011^T^.

The polar lipids of LD0937^T^ included diphosphatidylglycerol, phosphatidylglycerol, two unidentified amino phospholipids, eight unidentified glycolipids and one unidentified lipid (Fig. S6D). The polar lipids of SCR0063^T^ were different from those LD0937^T^ and contained an amino glycolipid, one more unidentified glycolipid and one more unidentified lipid (Fig. S6D and E). In contrast, *L. huelsenbergensis* DSM 115425^T^ contained diphosphatidylglycerol, phosphatidylglycerol, one aminoglycophospholipid, one unidentified amino glycolipid, one unidentified amino phospholipid, eight unidentified glycolipids, one unidentified phospholipid and four unidentified lipids (Fig. S6F). The polar lipids of *L. casei* DSM 20011^T^ included diphosphatidylglycerol, phosphatidylglycerol, two unidentified amino phospholipids, eight unidentified glycolipids and three unidentified lipids, a pattern similar to SCR0063^T^ (Fig. S6G). The polar lipid results showed that the two novel strains contained different polar lipids, but also had similar polar lipid compounds. The abovementioned results allow them to be clearly distinguished from each other and from the closest related strains *L. huelsenbergensis* DSM 115425^T^ and *L. casei* DSM 20011^T^.

All four investigated strains are facultatively heterofermentative and produce mainly l-lactic acid (more than 94 %), comparable to most representatives of the *Lacticaseibacillus* group [10,16, 17, 62].

Based on this polyphasic approach, using genomic, phylogenetic, phenotypic and chemotaxonomic analysis, LD0937^T^ and SCR0063^T^ could be clearly distinguished from their closest relatives and from each other. Strains LD0937^T^ and SCR0063^T^ should be classified as representing two novel species within the *Lacticaseibacillus* group named *Lacticaseibacillus parahuelsenbergensis* sp. nov. (type strain LD0937^T^=DSM 116105^T^=NCIMB 15471^T^) and *Lacticaseibacillus styriensis* sp. nov. (strain SCR0063^T^=DSM 116297^T^=NCIMB 15473^T^).

## Description of *Lacticaseibacillus zeae* subsp. *zeae* subsp. nov.

*Lacticaseibacillus zeae* subsp. *zeae* (zeʹae. L. n. *zea*, a kind of grain, and a botanical genus name; L. gen. n. *zeae*, of *Zea*, pertaining to *Zea mais*, corn).

*L. zeae* strains clustered in Group B (Table 1) belong to *L. zeae* subsp. *zeae*. Strains of this subspecies have dDDH values of 95.5–100 % with each other and dDDH values of 70.5–71.5 % with other *L. zeae* belonging to the subspecies *silagei*.

Rods are Gram-stain-positive, facultative heterofermentative, facultative anaerobic, non-motile and non-spore-forming with approximately 0.5‒0.6×1.2‒2.4 µm size. Colonies are approximately 1‒2 mm in diameter, smooth, glistening and white on MRS agar and can grow from 10 to 45 °C. Furthermore, growth occurs from pH 3.5 to 10.0 and with up to 8 % w/v NaCl. Cells are negative for nitrite and nitrate reduction, haemolysis, urease production, gas production from glucose, H_2_S production and indole production. A positive reaction is obtained for the Voges–Proskauer reaction. Enzyme activity is positive for alkaline phosphatase, acid phosphatase, esterase, esterase lipase, leucine arylamidase, valine aryl amidase, cystine aryl amidase, naphthol-AS-BI-phosphohydrolase, β-glucosidase, α-galactosidase, β-galactosidase and *N*-acetyl-β-glucosaminidase and negative for α-glucosidase, lipase, trypsin, α-chymotrypsin, β-glucuronidase, α-mannosidase and α-fucosidase. Acid is produced from ribose, galactose, d-glucose, d-fructose, d-mannose, rhamnose, mannitol, *N*-acetylglucosamine, amygdalin, arbutin, salicin, cellobiose, lactose, trehalose, sucrose, melezitose, β-gentiobiose, d-tagatose, l-fucose and gluconate. No acid is produced from sorbitol, maltose, d-arabitol, d-arabinose, l-arabinose, glycerol, adonitol, erythritol, dulcitol, inositol, d-xylose, l-xylose, methyl β-d-xylopyranoside, l-sorbose, methyl α-d-glucoside, methyl α-d-mannoside, melibiose, inulin, raffinose, starch, glycogen, xylitol, turanose, d-lyxose, d-fucose, l-arabitol, 2-keto gluconate and 5-keto gluconate.

The strain produces mainly l-lactic acid and a minor amount of d-lactic acid. The peptidoglycan type is A4α (l-Lys–d-Asp) in the presence of l-Lys, d-Asp, d-Ala and d-Glu. The major cellular fatty acids are C_18 : 1_ ω9*c*, summed feature 7 and C_16 : 0_. The major polar lipids are diphosphatidylglycerol, phosphatidylglycerol, amino phospholipid and glycolipids.

According to Rule 40b of the Bacteriological Code [61], the description of a new subspecies creates automatically the subspecies *Lacticaseibacillus zeae* subsp. *zeae* with the type strain DSM 20178^T^ (=ATCC 15820^T^=RIA 482^T^=CCUG 35515^T^=KCTC 3804^T^=JCM 11302^T^=BCRC 17942^T^=LMG 17315^T^=NCIMB 9537 ^T^).

The GenBank/EMBL/DDBJ accession numbers are D86516 (16S rRNA gene) and GCA_001433745.1 (draft genome), respectively.

## Description of *Lacticaseibacillus zeae* subsp. *silagei* subsp. nov.

*Lacticaseibacillus zeae* subsp. *silagei* (si.la'ge.i. N.L. gen. n. *silagei*, of silage).

Cells are facultative anaerobic, Gram-stain-positive, facultative heterofermentative, non-motile and non-spore-forming rods (0.6–0.8×2.0–2.5 µm). Colonies, grown for 48 h on MRS agar plates, are circular, cream, smooth, convex and approximately 1.1 mm in diameter. Cells can grow from 5 to 50 °C, at pH 3.5–9.0 and with 8.4 % w/v KCl, with optimal growth at 40 °C and pH 7.0. Tolerance of NaCl is up to 9 % w/v. Negative for nitrite and nitrate reduction, haemolysis, urease production, gas production from glucose, H_2_S production and indole production. A positive reaction is obtained for the Voges–Proskauer reaction. Enzyme activity is positive for esterase, esterase lipase, leucine aryl amidase, valine aryl amidase, acid phosphatase, naphthol-AS-BI-phosphohydrolase, α-glucosidase, β-glucosidase, α-galactosidase and β-galactosidase and negative for alkaline phosphatase, cystine aryl amidase, lipase, trypsin, α-chymotrypsin, β-glucuronidase, *N*-acetyl-β-glucosaminidase, α-mannosidase and α-fucosidase.

Acid is produced from d-arabinose, l-arabinose, ribose, galactose, d-glucose, d-fructose, d-mannose, rhamnose, mannitol, sorbitol, *N*-acetylglucosamine, amygdalin, arbutin, salicin, cellobiose, lactose, trehalose, maltose, sucrose, melezitose, β-gentiobiose, d-tagatose, d-arabitol, l-fucose and gluconate. No acid is produced from glycerol, adonitol, erythritol, dulcitol, inositol, d-xylose, l-xylose, methyl β-d-xylopyranoside, l-sorbose, methyl α-d-glucoside, methyl α-d-mannoside, melibiose, inulin, raffinose, starch, glycogen, xylitol, turanose, d-lyxose, d-fucose, l-arabitol, 2-keto gluconate and 5-keto gluconate. Both isomers of lactic acid are obtained (on average 3.5 % d-lactate and 96.5 % l-lactate). The peptidoglycan structure is of the l-Lys–d-Asp type in the presence of l-Lys, d-Asp and d-Glu. The major cellular fatty acids are C_16 : 0_, summed feature 7 and summed feature 8. The major polar lipids are diphosphatidylglycerol, phosphatidylglycerol, aminophospholipid, glycolipids and lipids.

The type strain EB0058^T^, isolated from Austrian corn silage, is deposited at the DSMZ – German Collection of Microorganisms (DSM 116376^T^) and the National Collections of Industrial Food and Marine Bacteria (NCIMB 15474^T^). The genome of the type strain consists of a circular chromosome with a size of 3 046 355 bp and a G+C content of 48.0 mol%. The genome sequence, the 16S rRNA sequence, and the raw read data of strain DSM 116376^T (^=NCIMB 15474^T^) are deposited at GenBank and SRA (accession numbers: GCF_030770335.1, OR603139, PRJNA1002954).

## Description of *Lacticaseibacillus parahuelsenbergensis* sp. nov.

*Lacticaseibacillus parahuelsenbergensis* (pa.ra.huel.sen.berg.en’sis. Gr. pref. *para*-, resembling; N.L. masc. adj. *huelsenbergensis*, a specific epithet referring to an area in the district of Bad Segeberg, Schleswig Holstein, Germany, company’s origin of ensiling research; N.L. masc. adj. *parahuelsenbergensis*, related to *Lacticaseibacillus huelsenbergensis*).

The cells are rod-shaped (0.8×2.0–2.5 µm), facultative anaerobic, facultative heterofermentative, Gram-stain-positive, non-motile and non-spore-forming. Colonies on MRS agar are circular with entire margins, creamy white, smooth, convex and approximately 1 mm in diameter after 2 days cultivation at 37 °C in aerobic conditions. Both l- (95 %) and d-lactate (5 %) are produced from glucose, while no gas is formed from glucose. Growth occurs with up to 8 % w/v NaCl. Cells can grow from 10 to 50 °C and pH 3.5 to 10.0, although 37 °C and pH 7.0 are the ideal growth conditions. Using the API 50 CHL system, acids are produced from d-arabinose, l-arabinose, adonitol, ribose, galactose, d-glucose, d-fructose, d-mannose, l-sorbose, rhamnose, inositol, mannitol, sorbitol, *N*-acetylglucosamine, amygdalin, arbutin, salicin, cellobiose, lactose, sucrose, trehalose, melezitose, β-gentiobiose, d-tagatose, l-fucose, l-arabitol and gluconate. No acid is obtained from dulcitol, glycerol, erythritol, turanose, d-xylose, l-xylose, methyl β-d-xylopyranoside, methyl α-d-glucoside, methyl α-d-mannoside, maltose, melibiose, inulin, raffinose, starch, glycogen, xylitol, d-lyxose, d-fucose, d-arabitol, 2-keto gluconate and 5-keto gluconate.

The strain is negative for haemolysis, urease production, H_2_S production, indole production, nitrite and nitrate reduction, and cell motility, while the Voges–Proskauer reaction leads to a positive result.

Enzyme activity is positive for esterase, lipase, leucine aryl amidase, valine aryl amidase, acid phosphatase, naphthol-AS-BI-phosphohydrolase, β-glucosidase, α-galactosidase and β-galactosidase and negative for α-glucosidase, alkaline phosphatase, lipase, trypsin, α-chymotrypsin, β-glucuronidase, *N*-acetyl-β-glucosaminidase, α-mannosidase, α-fucosidase and cystine arylamidase.

The major cellular fatty acids are C_14 : 0_, C_16 : 0_, C_18 : 1_ ω9*c* and summed feature 7. The peptidoglycan structure is of the l-Lys–d-Asp type in the presence of l-Lys, d-Asp and d-Glu. The polar lipids are composed of diphosphatidylglycerol, phosphatidylglycerol, amino phospholipids, glycolipids and lipids.

The type strain, LD0937^T^, isolated from an Austrian grass silage, is deposited at the German Collection of Microorganisms (DSM 116105^T^) and the National Collections of Industrial Food and Marine Bacteria (NCIMB 15471^T^). The genome of the type strain consists of a circular chromosome with a size of 3 055 873 bp and a G+C content of 48.0 mol%. The genome sequence, the 16S rRNA sequence, and the raw read data of strain DSM 116105^T^ are deposited at GenBank and SRA (accession numbers: GCA_030770265.1, OR603137, PRJNA1002950).

## Description of *Lacticaseibacillus styriensis* sp. nov.

*Lacticasibacillus styriensis* (sty.ri.en’sis. N.L. masc. adj. *styriensis*, with reference to Styria, federal state in Austria).

The cells are rod-shaped (0.9–1.0×2.5–3.0 µm), facultative anaerobic, Gram-stain-positive, non-motile, facultative heterofermentative and non-spore-forming. Colonies on MRS agar are circular with entire margins, white, smooth, convex and approximately 1.2 mm in diameter after 2 days cultivation at 37 °C in aerobic conditions. Both l- (94 %) and d-lactate (6 %) are produced from glucose and no gas is formed from glucose. Growth occurs with up to 8 % w/v NaCl. Cells can grow from 10 to 50 °C, with 8.4 % w/v KCl and from pH 3.5 to 10.0, although 40 °C and pH 7.0 are the ideal growth conditions. Acid is produced from d-arabinose, l-arabinose, adonitol, ribose, galactose, d-glucose, d-fructose, d-mannose, rhamnose, inositol, mannitol, sorbitol, methyl α-d-mannoside, *N*-acetylglucosamine, amygdalin, arbutin, salicin, cellobiose, lactose, sucrose, trehalose, β-gentiobiose, d-lyxose, turanose, d-tagatose, l-fucose, l-arabitol and gluconate. No acid is obtained from l-sorbose, dulcitol, glycerol, erythritol, d-xylose, l-xylose, methyl β-d-xylopyranoside, methyl α-d-glucoside, melezitose, maltose, melibiose, inulin, raffinose, starch, glycogen, xylitol, d-fucose, d-arabitol, 2-keto gluconate and 5-keto gluconate.

Urease production, H_2_S production, indole production, nitrite and nitrate reduction, haemolysis, and cell motility show negative results, while the Voges–Proskauer reaction is positive.

Enzyme activity is positive for alkaline phosphatase, esterase, lipase, leucine aryl amidase, valine aryl amidase, cystine aryl amidase, acid phosphatase, naphthol-AS-BI-phosphohydrolase, β-glucosidase, α-galactosidase, β-galactosidase and α-glucosidase and negative for lipase, trypsin, α-chymotrypsin, β-glucuronidase, *N*-acetyl-β-glucosaminidase, α-mannosidase and α-fucosidase.

The major cellular fatty acids are C_16 : 0_, C_18 : 1_ ω9*c* and summed feature 7. The peptidoglycan structure is of the l-Lys–d-Asp type in the presence of l-Lys, d-Asp and d-Glu. The polar lipids are composed of diphosphatidylglycerol, phosphatidylglycerol, amino phospholipids, amino glycolipids, glycolipids, and lipids.

The type strain, SCR0063^T^, isolated from Austrian corn silage, is deposited at the German Collection of Microorganisms (DSM 116297^T^) and the National Collections of Industrial Food and Marine Bacteria (NCIMB 15473^T^). The genome of the type strain consists of a circular chromosome with a size of 3 134 053 bp and a G+C content of 48.0 mol%. The genome sequence, the 16S rRNA sequence and the raw read data of strain DSM 116297^T^ are deposited at GenBank and SRA (accession numbers: GCA_030770295.1, OR603138, PRJNA1002952).

## supplementary material

10.1099/ijsem.0.006441 Uncited Supplementary Material 1.

## Abbreviations

AMR: antimicrobial resistance
ANI: average nucleotide identity
dDDH: digital DNA–DNA hybridization
MRS: de Man, Rogosa and Sharpe
