# Supplementary material for: Lacticaseibacillus parahuelsenbergensis sp. nov., Lacticaseibacillus styriensis sp. nov. and Lacticaseibacillus zeae subsp. silagei subsp. nov., isolated from different grass and corn silage
Source: Int J Syst Evol Microbiol. 2024 Jul 2;74(7):006441. doi: 10.1099/ijsem.0.006441 (PMC11316572; doi:10.1099/ijsem.0.006441)
Supplement: Uncited Supplementary Material 1. [file ijsem-74-06441-s001.pdf]

Supplementary Material

Lacticaseibacillus parahuelsenbergensis sp. nov., *Lacticaseibacillus styriensis* sp. nov. and *Lacticaseibacillus zeae* subsp. *silagei* subsp. nov., isolated from different grass and corn silage

Author names

Monika Grabner F.[1], Monika Grabner H.[1], Hermine Schein[1], Andrea Schrank[1], Manuela Töglhofer[1], Elisabeth Weidenholzer[1], Christian Rückert-Reed[2], Tobias Busche[3] and Marlene Buchebner-Jance[1]

Affiliation(s)

[1]Lactosan GmbH & Co.KG, Industriestraße West 5, 8605 Kapfenberg, Austria; [2]Technology Platform Genomics, CeBiTec, Bielefeld University, Germany; [3]Omics Core Facility NGS, Medical School OWL & CeBiTec, Bielefeld University, Germany.

Corresponding author and email address

Marlene Buchebner-Jance, buchebner@lactosan.at

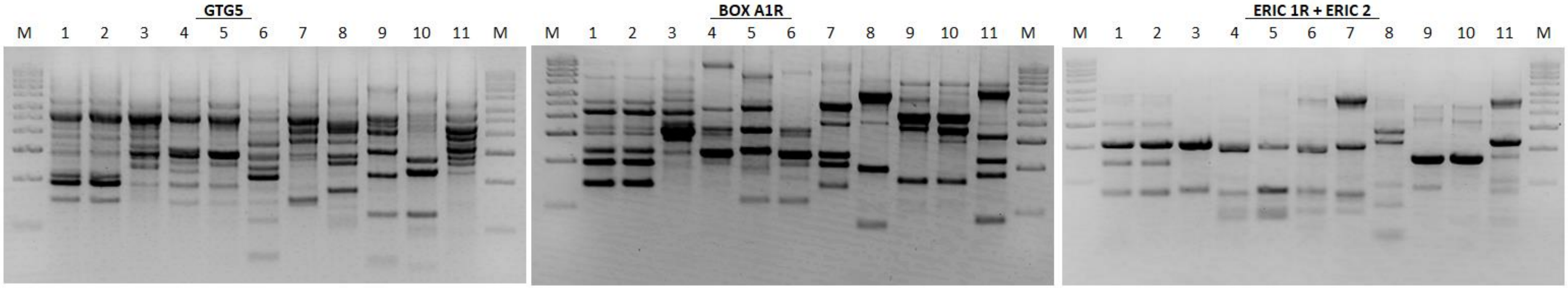

Supplementary Figure 1. Rep-PCR with the primers $(GTG)_5$, BOXA1R and ERIC1R + ERIC2. M, 1kb DNA marker (Roth); 1, EB0058^T; 2, SCR0080; 3, *L. zeae* DSM 20178^T; 4, LD 0937^T; 5, SCR0063^T; 6, *L. huelsenbergensis* DSM 115425^T; 7, *L. casei* DSM 20011^T; 8, *L. chiayiensis* NBRC112906^T; 9, *L. paracasei* subsp. *paracasei* DSM 5622^T; 10, *L. paracasei* subsp. *tolerans* DSM 20258^T; 11, *L. rhamnosus* DSM 20021^T.

Supplementary Table 1. BLAST analysis of the 16S rRNA, *argS*, *rpoA, pheS and rpoB* genes of EB0058^T, SCR0080, LD0937^T and SCR0063^T compared to all valid *Lacticaseibacillus* type strains currently available at LPSN, plus *L. zeae* genomes downloaded from NCBI.

	**16S rRNA**			**argS**	**rpoA**	**pheS**	**rpoB**
**Species (accession number)**	EB0058^T	LD0937^T	SCR0063^T	EB0058^T	EB0058^T	LD0937^T	SCR0063^T
***L. zeae* subsp. *silagei* EB0058^T (GCF_030770335.1)**	**100.0**	**99.5**	**99.4**	**100.0**	**100.0**	**96.3**	**97.1**
L. zeae subsp. *silagei* CECT9104 (GCF_900492555.1)	100.0	99.4	99.3	100.0	100.0	96.3	97.1
L. zeae subsp. *silagei* UD2202 (GCF_028878215.1)	100.0	99.4	99.3	100.0	100.0	96.3	97.1
***L. zeae* subsp. *silagei* SCR0080 (GCF_030770315.1)**	**100.0**	**99.5**	**99.4**	**100.0**	**100.0**	**96.3**	**97.1**
L. zeae subsp. *zeae* UHGG_MGYG-HGUT-02383 (GCF_902386575.1)	99.9	99.5	99.4	98.7	99.4	95.1	97.0
L. zeae subsp. *zeae* DSM 20178^T (GCF_001433745.1)	99.9	99.3	99.3	97.7	98.8	94.6	97.5
L. zeae subsp. *zeae* FBL8 (GCF_018363055.1)	99.9	99.5	99.4	97.9	98.9	94.7	97.2
L. zeae subsp. *zeae* KCTC 3804^T (GCF_000260435.1)	99.9	99.6	99.5	97.7	98.8	94.6	97.1
L. casei DSM 20011^T (GCF_000829055.1)	99.6	99.5	99.3	91.9	98.6	94.9	98.2
L. chiayiensis NBRC 112906^T (GCF_004123795.1)	99.6	99.5	99.3	80.0	98.9	90.4	94.7
L. huelsenbergensis DSM 115425^T (GCF_029726355.1)	99.6	99.5	99.4	92.3	99.0	96.4	98.4
***L. parahuelsenbergensis* LD0937^T (GCF_030770265.1)**	**99.6**	**100.0**	**99.4**	**92.4**	**98.5**	**100.0**	**98.5**
***L. styriensis* SCR0063^T (GCF_030770295.1)**	**99.6**	**99.4**	**100.0**	**92.0**	**98.6**	**96.9**	**100.0**
L. paracasei subsp. *tolerans* DSM 20258^T (GCF_001436485.1)	99.4	99.3	99.2	78.1	94.5	80.3	87.6
L. paracasei subsp. *paracasei* DSM 5622^T (GCF_001436385.1)	99.2	99.0	98.8	77.9	94.5	80.8	87.5
L. rhamnosus DSM 20021^T (GCF_001435405.1)	99.0	98.7	98.6	80.1	96.2	84.0	87.9
L. manihotivorans DSM 13343^T (GCF_001435035.1)	97.3	97.1	96.9	71.8	83.3	72.6	79.5
L. mingshuiensis 117-1^T (GCF_016861785.1)	96.4	96.2	96.1	71.4	81.2	70.3	78.8
L. yichunensis 33-1^T (GCF_003946405.1)	96.3	96.1	96.0	71.3	80.8	70.5	78.9

L. brantae DSM 23927T (GCF_001436115.1)	95.5	95.2	95.1	71.6	82.3	76.2	82.1
L. parakribbianus JCM 34954T (GCF 025490915.1)	95.5	95.2	95.0	71.1	79.9	69.6	79.0
L. saniviri DSM 24301T (GCF_001437465.1)	95.3	95.1	94.9	70.6	82.3	73.4	80.8
L. kribbianus YH-lac21T (GCF_020180945.1)	95.2	94.9	94.8	69.6	79.7	71.3	77.8
L. hegangensis 73-4T (GCF_003946465.1)	95.1	95.0	94.9	72.6	81.8	72.6	79.2
L. camelliae DSM 22697T (GCF_001436615.1)	95.0	94.8	94.6	72.0	82.0	71.7	79.5
L. suilingensis ZW152T (GCF_016861845.1)	94.9	94.7	94.6	72.1	81.7	73.7	81.7
L. baoqingensis 47-3T (GCF_003946165.1)	94.9	94.7	94.6	72.3	82.1	73.9	79.3
L. suibinensis 247-3T (GCF_003946175.1)	94.9	94.7	94.6	71.5	81.4	73.3	81.5
L. porcinae JCM 19617T (GCF_003946305.1)	94.8	94.6	94.5	72.6	83.3	72.4	79.6
L. jixianensis 159-4T (GCF_003946485.1)	94.6	94.4	94.2	70.9	80.6	72.0	80.0
L. absianus YH-lac23T (GCF_013407595.1)	94.5	94.2	94.1	69.9	81.0	71.9	78.1
L. daqingensis 143-4(a)T (GCF_003946115.1)	94.3	94.1	94.0	70.6	80.3	73.2	79.4
L. nasuensis JCM 17158T (GCF_001434705.1)	94.2	94.0	93.9	71.1	79.7	74.4	80.7
L. zhaodongensis 1206-1T (GCF_009687905.1)	94.2	94.1	93.9	69.5	77.6	69.4	78.3
L. songhuajiangensis 7-19T (GCF_003946025.1)	93.8	93.5	93.4	69.3	75.5	70.5	78.6
L. pantheris NBRC 106106T (GCF_001591865.1)	93.6	93.4	93.3	68.4	73.9	70.3	78.3
L. thailandensis DSM 22698T (GCF_001436135.1)	93.5	93.4	93.2	69.9	73.3	71.1	78.4
L. sharpeae DSM 20505T (GCF_001436225.1)	93.3	93.0	93.0	70.7	77.6	70.6	77.9
L. hulanensis ZW163T (GCF_004010095.1)	92.8	92.6	92.4	70.2	77.3	69.4	78.0

Supplementary Table 2. Assembly statistics of the whole genome sequence assembly of 1, EB0058[T], 2, SCR0080; 3, LD0937[T]; 4, SCR0063[T].

	1	2	3	4
accession number	GCF_030770335.1	GCF_030770315.1	GCF_030770265.1	GCF_030770295.1
genome size	3,046,355	3,073,808	3,055,873	3,134,053
N50	3,046,355	3,073,808	3,055,873	3,134,053
G and C content [ml%]	48	48	48	48
coverage total	941	281	525	381
	1 contig per replicon. Here 1 chromosome, no plasmids.	1 contig per replicon. Here 1 chromosome, no plasmids.	1 contig per replicon. Here 1 chromosome, no plasmids.	1 contig per replicon. Here 1 chromosome, no plasmids.
Illumina Data				
coverage	382	144	318	178
reads	4,112,843	1,544,789	3,403,756	1,860,046
bases	1,162,770,208	441,541,786	972,540,631	559,328,047
ONT Data				
coverage	559	137	207	203
reads	369,785	96,965	150,749	148,533
bases	1,703,400,428	420,920,939	633,187,301	635,533,168
prokka annotation<br>number of annotated features				
CDS	2777	2824	2792	2906
unique / gene codes	1156	1156	1177	1189
CRISPR	3	3	0	0
ncRNAs	33	32	28	28
rRNAs	15	15	15	15
tRNAs	60	60	60	61
pgap annotation (2023-05-17.build6771)				
Genes	2825	2872	2846	2931
protein-conding	2723	2764	2752	2830
Quality analysis (checkm v1.2.2)				
Completeness	99.35	99.35	99.35	99.35
Contamination	0.76	0.76	0.76	0.11

Supplementary Table 3. Average Nucleotide Identity (ANI) values and digital DNA-DNA-hybridisation (dDDH, formula 2) values for EB0058^T, SCR0080, LD0937^T and SCR0063^T compared to all valid *Lacticaseibacillus* type strains currently available at LPSN, plus *L. zeae* genomes downloaded from NCBI.

	dDDH			ANI		
**Species (accession number)**	EB0058^T	LD 0937^T	SCR0063^T	EB0058^T	LD 0937^T	SCR0063^T
***L. zeae* subsp. *silagei* EB0058^T (GCF_030770335.1)**	**100.0**	**54.2**	**54.8**	100.0	93.7	93.6
***L. zeae* subsp. *silagei* SCR0080 (GCF_030770315.1)**	**99.6**	**54.1**	**54.7**	99.9	93.7	93.6
L. zeae subsp. *silagei* CECT9104 (GCF_900492555.1)	99.3	54.2	54.8	99.9	93.7	93.6
L. zeae subsp. *silagei* UD2202 (GCF_028878215.1)	99.3	53.8	54.5	99.9	93.7	93.6
L. zeae subsp. *zeae* UHGG_MGYG-HGUT-02383 (GCF_902386575.1)	72.5	53.9	54.5	96.6	93.7	93.7
L. zeae subsp. *zeae* FBL8 (GCF_018363055.1)	71.5	54.4	55.0	96.4	93.6	93.5
L. zeae subsp. *zeae* DSM 20178^T (GCF_001433745.1)	70.8	53.9	54.6	96.4	93.6	93.5
L. zeae subsp. *zeae* KCTC 3804^T (GCF_000260435.1)	70.8	54.1	54.8	96.4	93.6	93.5
L. casei DSM 20011^T (GCF_000829055.1)	57.1	64.1	64.9	93.7	95.4	95.5
***L. styriensis* SCR0063^T (GCF_030770295.1)**	**54.8**	**67.6**	**100.0**	93.6	95.9	100.0
L. huelsenbergensis DSM 115425^T (GCF_029726355.1)	54.3	66.5	65.9	93.8	95.7	95.6
***L. parahuelsenbergensis* LD0937^T (GCF_030770265.1)**	**54.2**	**100.0**	**67.6**	93.6	100.0	95.9
L. chiayiensis NBRC 112906^T (GCF_004123795.1)	35.8	35.5	35.7	87.8	88.0	88.1
L. zhaodongensis 1206-1^T (GCF_009687905.1)	32.1	24.5	24.6	67.7	67.4	67.5
L. manihotivorans DSM 13343^T (GCF_001435035.1)	27.4	24.9	26.1	68.7	68.2	68.2
L. kribbianus YH-lac21^T (GCF_020180945.1)	26.9	26.0	26.8	68.4	68.1	68.1
L. suibinensis 247-3^T (GCF_003946175.1)	26.3	20.8	21.4	69.7	69.0	68.9
L. thailandensis DSM 22698^T (GCF_001436135.1)	24.8	25.4	24.9	67.3	67.2	67.3
L. songhuajiangensis 7-19^T (GCF_003946025.1)	24.2	23.9	24.0	67.6	67.4	67.2

L. sharpeae DSM 20505^{T} (GCF_001436225.1)	24.0	23.9	22.9	67.6	67.6	67.7
L. rhamnosus DSM 20021^{T} (GCF_001435405.1)	23.9	23.7	23.7	78.7	78.7	78.6
L. porcinae JCM 19617^{T} (GCF_003946305.1)	23.8	24.5	25.1	68.4	68.3	68.4
L. brantae DSM 23927^{T} (GCF_001436115.1)	23.6	21.4	23.5	68.9	68.8	68.8
L. absianus YH-lac23^{T} (GCF_013407595.1)	23.4	24.0	24.8	68.3	67.7	67.6
L. hegangensis 73-4^{T} (GCF_003946465.1)	23.3	23.0	23.0	69.0	68.0	68.1
L. pantheris NBRC 106106^{T} (GCF_001591865.1)	23.3	24.2	23.2	67.3	67.2	67.1
L. parakribbianus JCM 34954^{T} (GCF 025490915.1)	23.2	23.7	24.2	68.7	68.4	68.4
L. baoqingensis 47-3^{T} (GCF_003946165.1)	23.1	21.9	21.6	69.2	68.4	68.4
L. mingshuiensis 117-1^{T} (GCF_016861785.1)	23.1	22.0	22.6	68.9	68.6	68.6
L. hulanensis ZW163^{T} (GCF_004010095.1)	23.0	24.1	24.8	67.9	67.5	67.4
L. camelliae DSM 22697^{T} (GCF_001436615.1)	22.9	21.6	22.4	68.8	68.0	68.0
L. paracasei subsp. *tolerans* DSM 20258^{T} (GCF_001436485.1)	22.9	22.7	22.9	77.2	77.3	77.4
L. daqingensis 143-4(a)^{T} (GCF_003946115.1)	22.8	21.5	23.1	68.5	68.0	68.0
L. paracasei subsp. *paracasei* DSM 5622^{T} (GCF_001436385.1)	22.8	22.7	22.8	77.1	77.2	77.2
L. saniviri DSM 24301^{T} (GCF_001437465.1)	22.7	22.6	23.1	68.9	68.7	68.7
L. yichunensis 33-1^{T} (GCF_003946405.1)	22.4	22.6	22.8	68.7	68.5	68.6
L. suilingensis ZW152^{T} (GCF_016861845.1)	22.2	21.7	22.3	69.0	69.0	68.9
L. jixianensis 159-4^{T} (GCF_003946485.1)	21.6	22.5	22.2	68.5	68.1	68.1
L. nasuensis JCM 17158^{T} (GCF_001434705.1)	21.4	21.0	21.3	68.6	68.5	68.5

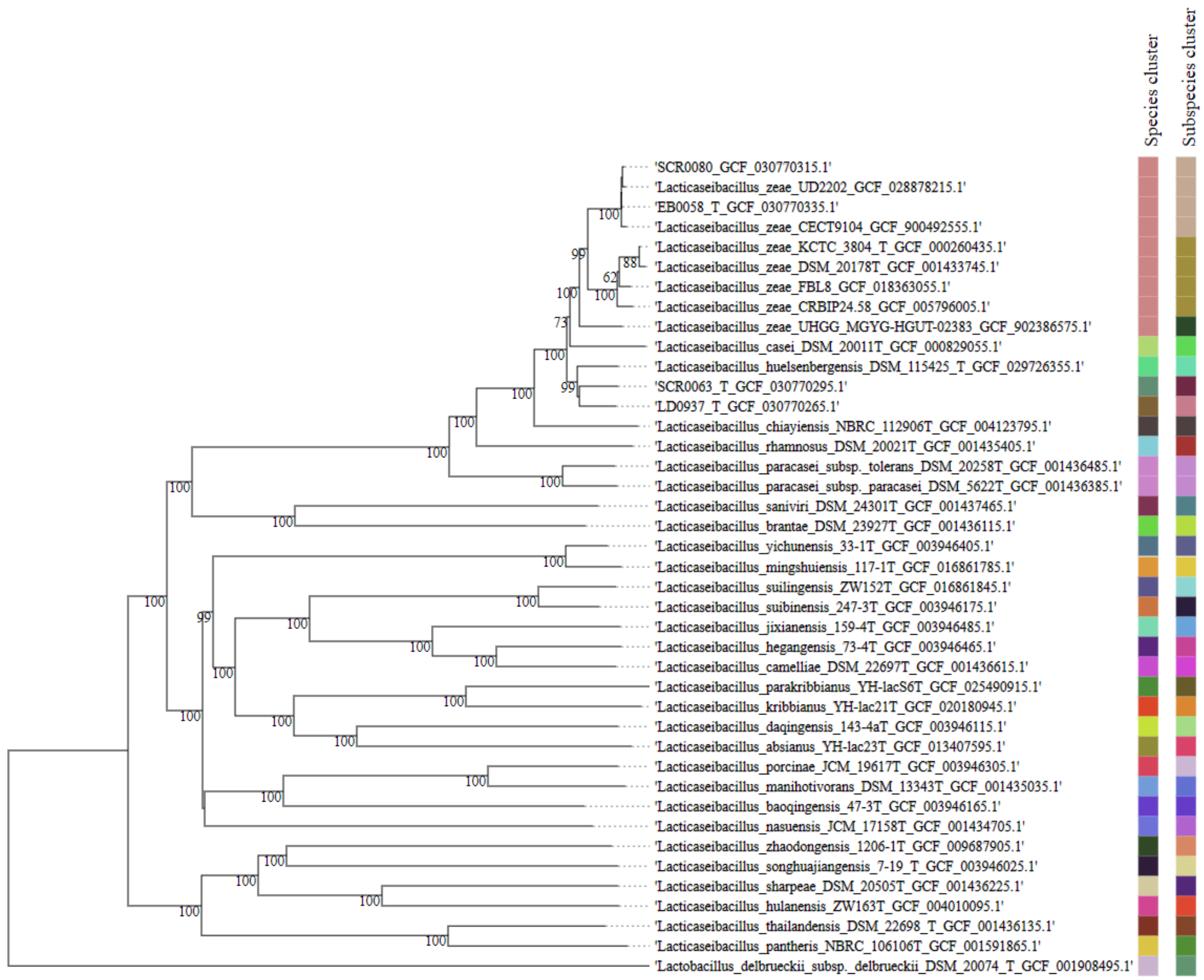

Supplementary Figure 2. Phylogenetic analysis of the novel isolates' EB0058^{T}, SCR0080, LD0937^{T} and SCR0063^{T} core genome using the proteome based TYGS phylogeny. Reference strains are all valid *Lacticaseibacillus* type strains currently available at LPSN, plus *L. zeae* genomes downloaded from ncbi, *Lactobacillus delbrueckii* subsp. *delbrueckii* DSM 20074^{T} served as outgroup. The heatmap on the right shows the classification into species or subspecies clusters. Tree inferred with FastME 2.1.6.1 (62) from whole-proteome-based GBDP distances. The branch lengths are scaled via GBDP distance formula d_5. Branch values are GBDP pseudo-bootstrap support values > 60% from 100 replications, with an average branch support of 91.3%. The tree was midpoint-rooted (63).

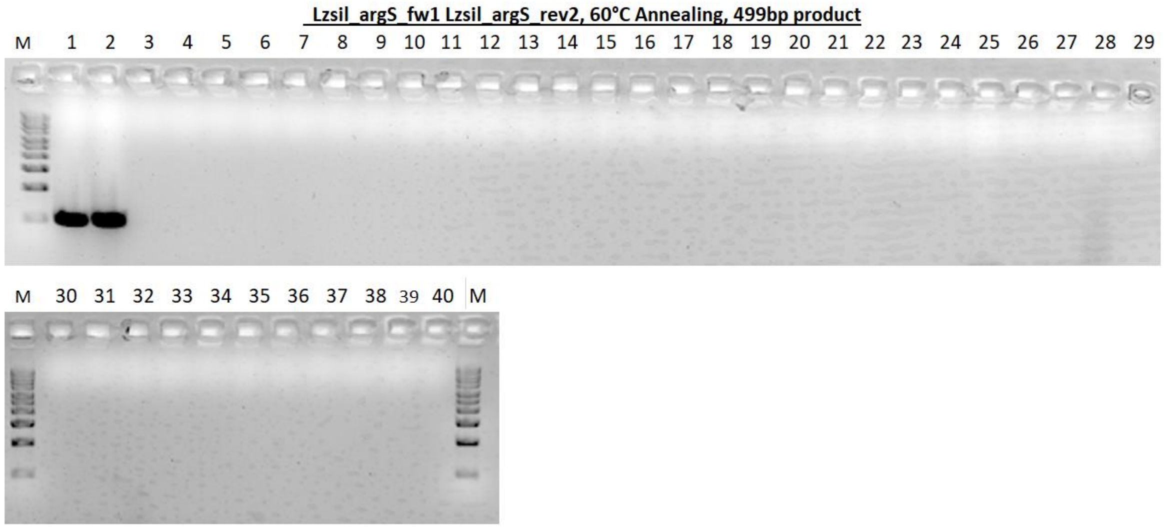

Supplementary Figure 3. PCR with primers specifically amplifying the partial *argS* gene of *L. zeae* subsp. *silagei*. Primers were Lzsil_*argS*_fw1 and Lzsil_*argS*_rev2, the annealing temperature was 60°C. The size of the expected product is 499bp. M, 1kb DNA marker (Roth); 1, EB0058^{T}; 2, SCR0080; 3, *L. zeae* DSM 20178^{T} ; 4, LD0937^{T}; 5, SCR0063^{T}; 6, *L. huelsenbergensis* DSM 115425^{T} ; 7, *L. casei* DSM 20011^{T} ; 8, *L. chiayiensis* NBRC112906^{T} ; 9, *L. paracasei* subsp. *paracasei* DSM 5622^{T} ; 10, *L. paracasei* subsp. *tolerans* DSM 20258^{T} ; 11, *L. rhamnosus* DSM 20021^{T}; 12, *S. perolens* DSM 12744^{T}; 13, *L. yichunensis* NCIMB 15169^{T}; 14, *L. diolivorans* DSM 14221^{T}; 15, *L. reuteri* DSM 20016^{T}; 16, *L. sakei* subsp. *sakei* DSM 20017^{T}; 17, *L. buchneri* subsp. *buchneri* DSM 20057^{T}; 18, *L. brevis* DSM 20054^{T}; 19, *L. cremoris* subsp. *cremoris* DSM 20069^{T}; 20, *L. lactis* subsp. *lactis* DSM 20481^{T}; 21, *L. delbrueckii* subsp. *bulgaricus* DSM 20081^{T}; 22, *L. farraginis* DSM 18382^{T}; 23, *L. parafarraginis* DSM 18390^{T}; 24, *L. curvatus* DSM 20019^{T}; 25, *L. plantarum* subsp. *plantarum* DSM 20174^{T}; 26, *F. sanfransiscensis* DSM 20451^{T}; 27, *L. amylovorus* DSM 20531^{T}; 28, *L. fermentum* DSM 20052^{T}; 29, *S. collinoides* DSM 20515^{T}; 30, *L. acidophilus* DSM 20079^{T}; 31, *L. frumenti* DSM 13145^{T}; 32, *L. kefiri* DSM 20587^{T}; 33, *L. vaginalis* DSM 5837T; 34, *L coryniformis* subsp. *coryniformis* DSM 20001T; 35, *L. rapi* DSM 19907^{T}; 36, *L. pentosus* DSM 20314^{T}; 37, *L. kisonensis* DSM 19906^{T}; 38, *L. hilgardii* DSM 20176^{T}; 39, *L. parabuchneri* DSM 5707^{T}; 40, *L. gasseri* DSM 25908^{T}.

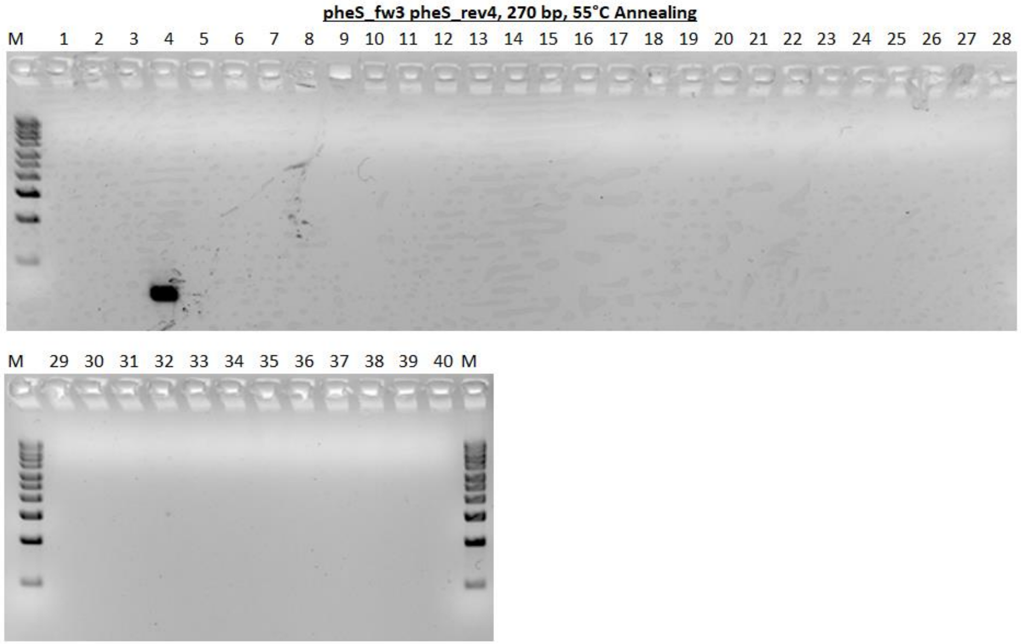

Supplementary Figure 4. PCR with primers specifically amplifying the partial *pheS* gene of *L. parahuelsenbergensis*. Primers were phe*S*_fw3 and phe*S*_rev4, the annealing temperature was 55°C. The size of the expected product is 270bp. M, 1kb DNA marker (Roth); 1, EB0058^{T}; 2, SCR0080; 3, *L. zeae* DSM 20178^{T} ; 4, LD0937^{T}; 5, SCR0063^{T}; 6, *L. huelsenbergensis* DSM 115425^{T} ; 7*, L. casei* DSM 20011^{T} ; 8, *L. chiayiensis* NBRC112906^{T} ; 9, *L. paracasei* subsp. *paracasei* DSM 5622^{T} ; 10*, L. paracasei* subsp. *tolerans* DSM 20258^{T} ; 11, *L. rhamnosus* DSM 20021^{T}; 12*, S. perolens* DSM 12744^{T}; 13, *L. yichunensis* NCIMB 15169^{T}; 14, *L. diolivorans* DSM 14221^{T}; 15, *L. reuteri* DSM 20016^{T}; 16, *L. sakei* subsp. *sakei* DSM 20017^{T}; 17, *L. buchneri* subsp. *buchneri* DSM 20057^{T}; 18, *L. brevis* DSM 20054^{T}; 19, *L. cremoris* subsp. *cremoris* DSM 20069^{T}; 20*, L. lactis* subsp. *lactis* DSM 20481^{T}; 21, *L. delbrueckii* subsp. *bulgaricus* DSM 20081^{T}; 22, *L. farraginis* DSM 18382^{T}; 23*, L. parafarraginis* DSM 18390^{T}; 24, *L. curvatus* DSM 20019^{T}; 25*, L. plantarum* subsp. *plantarum* DSM 20174^{T}; 26, *F. sanfransiscensis* DSM 20451^{T}; 27*, L. amylovorus* DSM 20531^{T}; 28, *L. fermentum* DSM 20052^{T}; 29, *S. collinoides* DSM 20515^{T}; 30*, L. acidophilus* DSM 20079^{T}; 31, *L. frumenti* DSM 13145^{T}; 32, *L. kefiri* DSM 20587^{T}; 33, *L. vaginalis* DSM 5837T; 34, *L coryniformis* subsp. *coryniformis* DSM 20001T; 35, *L. rapi* DSM 19907^{T}; 36*, L. pentosus* DSM 20314^{T}; 37, *L. kisonensis* DSM 19906^{T}; 38*, L. hilgardii* DSM 20176^{T}; 39, *L. parabuchneri* DSM 5707^{T}; 40, *L. gasseri* DSM 25908^{T}.

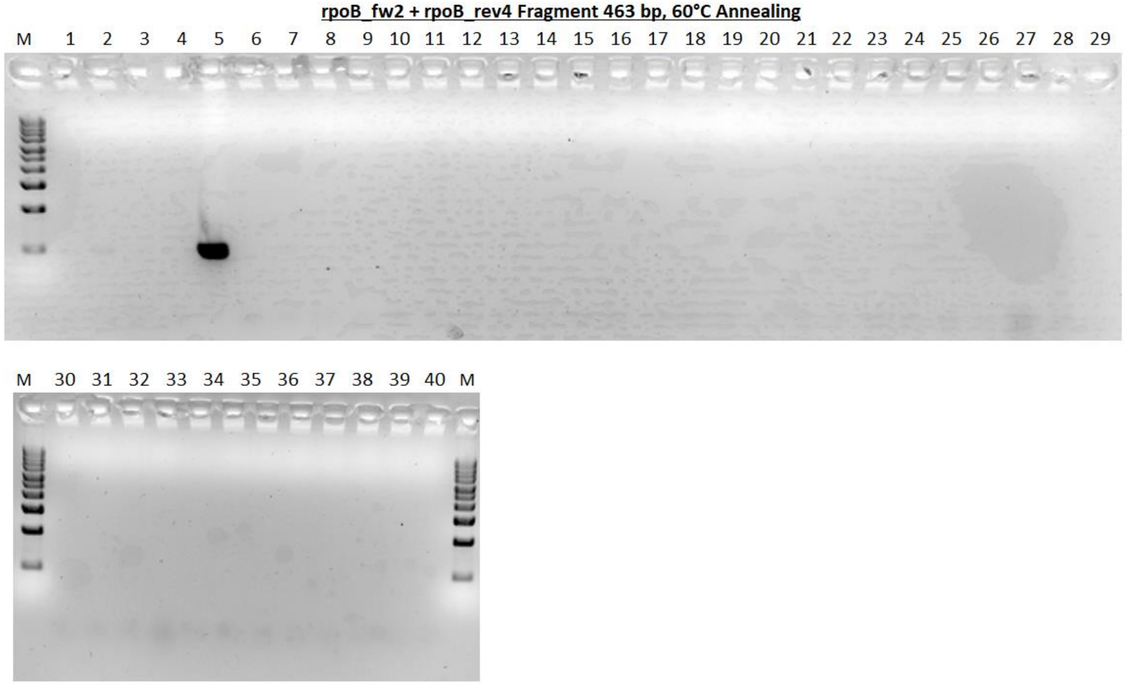

Supplementary Figure 5. PCR with primers specifically amplifying the partial *rpoB* gene of *L. styriensis*. Primers were rpoB_fw2 and rpoB_rev4, the annealing temperature was 60°C. The size of the expected product is 463bp. M, 1kb DNA marker (Roth); 1, EB0058^{T}; 2, SCR0080; 3, *L. zeae* DSM 20178^{T} ; 4, LD0937^{T}; 5, SCR0063^{T}; 6, *L. huelsenbergensis* DSM 115425^{T} ; 7, *L. casei* DSM 20011^{T} ; 8, *L. chiayiensis* NBRC112906^{T} ; 9, *L. paracasei* subsp. *paracasei* DSM 5622^{T} ; 10, *L. paracasei* subsp. *tolerans* DSM 20258^{T} ; 11, *L. rhamnosus* DSM 20021^{T}; 12, *S. perolens* DSM 12744^{T}; 13, *L. yichunensis* NCIMB 15169^{T}; 14, *L. diolivorans* DSM 14221^{T}; 15, *L. reuteri* DSM 20016^{T}; 16, *L. sakei* subsp. *sakei* DSM 20017^{T}; 17, *L. buchneri* subsp. *buchneri* DSM 20057^{T}; 18, *L. brevis* DSM 20054^{T}; 19, *L. cremoris* subsp. *cremoris* DSM 20069^{T}; 20, *L. lactis* subsp. *lactis* DSM 20481^{T}; 21, *L. delbrueckii* subsp. *bulgaricus* DSM 20081^{T}; 22, *L. farraginis* DSM 18382^{T}; 23, *L. parafarraginis* DSM 18390^{T}; 24, *L. curvatus* DSM 20019^{T}; 25, *L. plantarum* subsp. *plantarum* DSM 20174^{T}; 26, *F. sanfransiscensis* DSM 20451^{T}; 27, *L. amylovorus* DSM 20531^{T}; 28, *L. fermentum* DSM 20052^{T}; 29, *S. collinoides* DSM 20515^{T}; 30, *L. acidophilus* DSM 20079^{T}; 31, *L. frumenti* DSM 13145^{T}; 32, *L. kefiri* DSM 20587^{T}; 33, *L. vaginalis* DSM 5837T; 34, *L coryniformis* subsp. *coryniformis* DSM 20001T; 35, *L. rapi* DSM 19907^{T}; 36, *L. pentosus* DSM 20314^{T}; 37, *L. kisonensis* DSM 19906^{T}; 38, *L. hilgardii* DSM 20176^{T}; 39, *L. parabuchneri* DSM 5707^{T}; 40, *L. gasseri* DSM 25908^{T}.

Supplementary Table 4. Number of coding sequences (CDS) of various KEGG Pathways of the novel strains and the closest related species.

Strains: 1, EB0058^T; 2, SCR0080; 3, *L. zeae* DSM 20178^T; 4, LD0937^T; 5, SCR0063^T; 6*, L. huelsenbergensis* DSM 115425^T.

Pathway (KEGG No.)	1	2	3	4	5	6
ABC transporters (ko02010)	90	89	78	77	81	80
Ascorbate and aldarate metabolism (ko00053)	3	3	2	8	8	8
Biosynthesis of secondary metabolites (ko01110)	154	155	154	164	170	158
Carbon metabolism (ko01200)	52	53	50	54	54	54
Fructose and mannose metabolism (ko00051)	26	26	28	29	31	26
Galactose metabolism (ko00052)	32	32	29	29	33	33
Glycine, serine and threonine metabolism (ko00260)	12	13	13	15	18	14
Glycolysis / Gluconeogenesis (ko00010)	23	24	25	28	28	25
Metabolic pathways (ko01100)	402	404	400	418	432	412
Microbial metabolism in diverse environments (ko01120)	99	100	97	108	117	112
Pentose and glucuronate interconversions (ko00040)	14	14	13	19	20	19
Pentose phosphate pathway (ko00030)	25	25	20	25	27	25
Phosphotransferase system PTS (ko02060)	37	37	35	40	45	37
Starch and sucrose metabolism (ko00500)	23	23	24	25	26	22
Two-component system (ko02020)	45	45	46	47	46	46

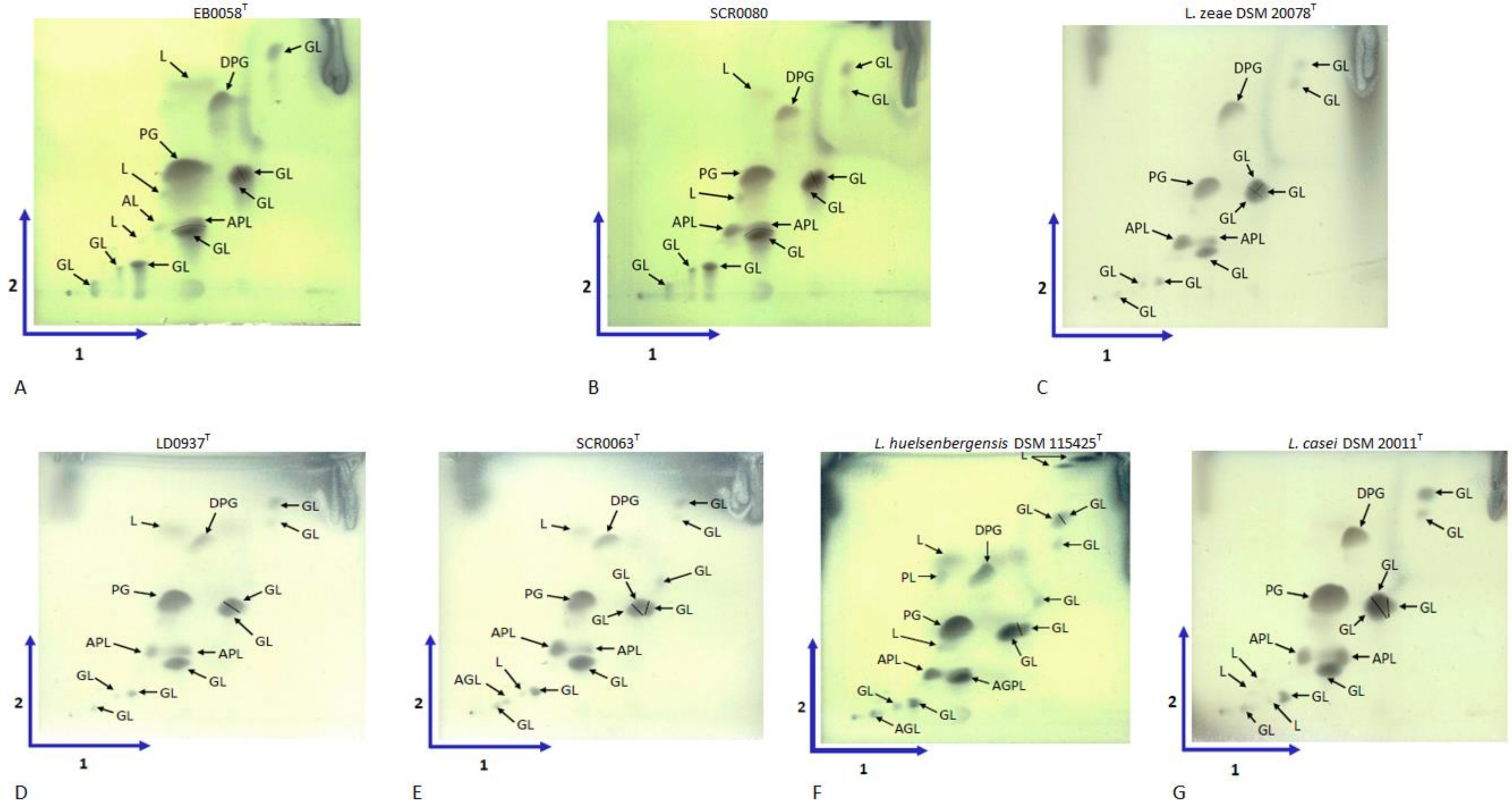

Supplementary Figure 6. Analysis of the polar lipids of A, EB0058^T; B, SCR0080; C, *L. zeae* DSM 20178^T; D, LD0937^T; E, SCR0063^T; F, *L. huelsenbergensis* DSM 115425^T; G, *L. casei* DSM 20011^T. AL, Aminolipid; AGL, Aminoglycolipid; AGPL, Aminoglycophospholipid; APL, Aminophospholipid; DPG, Diphosphatidylglycerol; GL, Glycolipid; L, Lipid; PG, Phosphatidylglycerol; PL, Phospholipid.
